# Solar Power Prediction Using Dual Stream CNN-LSTM Architecture

**DOI:** 10.3390/s23020945

**Published:** 2023-01-13

**Authors:** Hamad Alharkan, Shabana Habib, Muhammad Islam

**Affiliations:** 1 Department of Electrical Engineering, Unaizah College of Engineering, Qassim University, Unaizah 56452, Saudi Arabia; 2 Department of Information Technology, College of Computer, Qassim University, Buraydah 51452, Saudi Arabia; 3 Department of Electrical Engineering, College of Engineering and Information Technology, Onaizah Colleges, Onaizah 56447, Saudi Arabia

**Keywords:** solar power prediction, CNN, LSTM, dual-stream network

## Abstract

The integration of solar energy with a power system brings great economic and environmental benefits. However, the high penetration of solar power is challenging due to the operation and planning of the existing power system owing to the intermittence and randomicity of solar power generation. Achieving accurate predictions for power generation is important to provide high-quality electric energy for end-users. Therefore, in this paper, we introduce a deep learning-based dual-stream convolutional neural network (CNN) and long short-term nemory (LSTM) network followed by a self-attention mechanism network (DSCLANet). Here, CNN is used to learn spatial patterns and LSTM is incorporated for temporal feature extraction. The output spatial and temporal feature vectors are then fused, followed by a self-attention mechanism to select optimal features for further processing. Finally, fully connected layers are incorporated for short-term solar power prediction. The performance of DSCLANet is evaluated on DKASC Alice Spring solar datasets, and it reduces the error rate up to 0.0136 MSE, 0.0304 MAE, and 0.0458 RMSE compared to recent state-of-the-art methods.

## 1. Introduction

Regarding solar energy generation, sustainable development and global climate change are the two main issues [1]. Each year energy consumption is increased by 2% globally, where the total energy production is significantly based on fossil fuels, such as natural gas, coal, and oil, which considerably increases anthropogenic greenhouse gas (GHG) emission [2,3]. Furthermore, power generation from fuels produces environmental risks and energy crises, such as energy resource reduction and an increase in environmental pollution, which is considered a major threat to lives [4,5,6]. These drawbacks of energy generation from fossil fuels force governments to explore the resources of renewable energies [6,7].

Solar power is considered the alternative when compared to fossil fuels due to various characteristics, such as being clean, green, and naturally replenished. Solar power generation, either as an islanded or grid-connected mode of operation, brings unstable uncertainty, which causes problems for the stability of the power systems, particularly for the integration of solar power in a large microgrid system [8,9]. To overcome these challenges a reliable solar power prediction is an effective way to decrease the uncertainty, which is important for the planning, management, and operation of energy systems [10]. Therefore, the researchers investigated several techniques for solar power prediction. These techniques are broadly categorized into statistical (ST), artificial intelligence (AI), and hybrid methods (HM) [11]. In ST-based methods, several algorithms are developed, including auto-regressive [12], Bayesian [13], Kalman [14], grey models [15,16], and the Markov chain model [17]. Additionally, MaatAllah et al. [18] and Reikard et al. [19] developed ST-based models for renewable power prediction. In contrast, statistical models rely on linear data for learning but are unable to learn complex data; therefore, ST-based methods are not recommended for problems requiring nonlinear predictions, such as those associated with solar power.

Due to their potential for extracting representative features and data mining, AI-based models have proven to be more successful than physical and statistical ones [20]. These AI-based methods developed in the literature for solar power generation include neural networks [21], SVR [22], the adaptive fuzzy approach [23], and ELM [24], etc. Unlike ST-based approaches, most of these AI-based approaches are used to manage nonlinear relationships between input and output. Additionally, in the literature on power generation prediction, some special AI-based models, such as those models based on CNNs and generative adversarial networks, were developed by [25], and it became evident that weather classification played a significant role in developing such an accurate model. Furthermore, a number of AI-based approaches, including RNN [26], LSTM [27], CNN [28], GRU [29], etc., have been developed by the researchers for solar power generation, where the details are given in a recent survey [30]. This survey [30] also concluded that due to balancing parameter stability with accuracy, and their pros and cons, hybrid models are effective for solar power prediction. These AI-based methods are constructed via shallow architecture, requiring handcrafted feature engineering and having limited generalization capabilities [31]. Furthermore, in AI-based methods, CNN and RNNs achieved better performance; however, using CNN, the feature is extracted in spatial dimensions [32,33,34], while the RNNS learns in temporal dimensions, while solar power generation includes both types of features. Therefore, an approach with the ability of spatial and temporal feature extraction is required for accurate solar power prediction.

**Table 1:** Summary of hybrid methods developed for power generation prediction.

Ref.	Method	Comparison	Summary
Agoua et al. [35]	Spatiotemporal network	Auto-regression and decision tree	A spatiotemporal network is developed for learning spatial and temporal information.
Gensler et al. [36]	Auto-LSTM	MLP, ANN, LSTM, DNN, DBN	Developed an LSTM- and MLP-based hybrid model.
Sorkun et al. [37]	LSTM	LSTM, naive, GRU, RNN, and LSTM	Developed an LSTM-based method for power generation forecasting.
Khan et al. [38]	CNNESN	LSTM, GRU, ESN	A combined CNN- and ESN-based model is developed.
Dey et al. [39]	SolarNet	Gaussian regression, SVR, ANN	A CNN-based model for power generation prediction is developed.
Abdel et al. [26]	LSTMRNN	ANN and regression	A RNN-LSTM-based hybrid model is developed.
Khan et al. [38]	CNNESN	SVR, decision tree, CNN, LSTM	A combined CNN- and ESN-based model is developed.
Yan et al. [40]	CNN-GRU	LSTM and GRU	A combined inception and GRU model.
Dong et al. [41]	chaotic hybrid CNN model	CNN-based ablation study	The performance of a CNN-based model was developed and improved their performance with the use of a chaotic hybrid model.
Khan et al. [7]	ESN-CNN	Detailed ablation study	Integrated ESN and CNN for power generation prediction

In the light of current literature, hybrid models achieved state-of-the-art accuracy for solar power prediction [38]. These models include CNN-RNN [42], CNN-GRU [43], CNN-LSTM [44], CNNLSTM with autoencoder [45], convolutional LSTM (CLSTM) [46], CNN-GRU with preprocessing [45,47], and LSTM-CNN [48]. Some recent hybrid models for renewable power generation prediction are summarized in Table 1. Hybrid methods achieved improved prediction performance compared to other predictive modeling techniques. However, the current literature focuses on the stacked layers procedure to develop a hybrid model for solar power prediction where historical data of solar power have a limited number of features, which makes it difficult to learn spatial and temporal features using the stacked layers phenomena. Furthermore, prediction accuracy needs to be improved for reliable and accurate solar energy prediction. Therefore, in this work, we developed DSCLANet for solar power prediction with the ability to learn spatial and temporal features parallelly from actual solar power and weather data. The first stream of the proposed network utilizes CNN for spatial feature extraction, while the second stream is responsible for temporal feature extraction. Finally, the outcome of these streams is concatenated and passed to fully connected layers for solar energy prediction. The performance of the proposed model is evaluated on benchmark datasets and extensively decreases the error rates compared to state-of-the-art models. The following are the main contributions of this work:

- To select the most suitable model for solar power prediction, an ablation study is conducted, where the main objective is to evaluate the performance of several techniques including CNN, LSTM, GRU, CNNLSTM, CNNGRU, and DSCLANet to select an accurate prediction model for solar power.
- Our findings from this ablation study indicate that DSCLANet gives the best prediction accuracy comparatively, which has been confirmed experimentally by various comparisons. The DSCLANet process is the input via separate streams for spatial and temporal features which are then fused and passed to the attention for feature refinement. The refined features are then forwarded to a fully connected layer for final solar power prediction.
- A number of benchmark datasets are utilized to assess the DSCLANet performance, and the results indicate a marginal reduction in error rates compared to other state-of-the-art methods.
- The remainder of this article is organized as follows. Section 2 describes the internal architecture of DSCLANet, and Section 3 defines the datasets, evaluation metrics, and performance comparison of DSCLANet with ablation study and baseline methods. Finally, this article is concluded in Section 4, with possible future directions.

## 2. Materials and Methods

**Figure 1:**
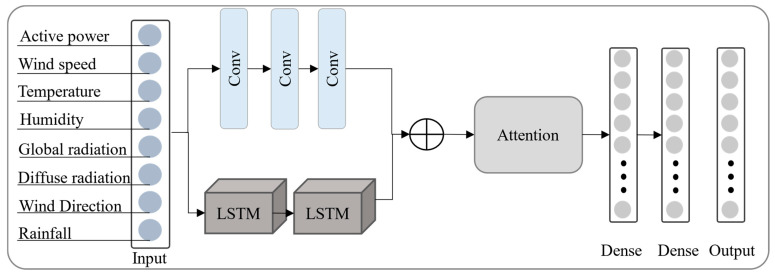
The proposed DSCLANet framework for solar power prediction.

The main framework of the DSCLANet is shown in Figure 1. where the input data is parallelly processed using CNN and LSTM architecture to extract spatiotemporal information. The output of these two architectures is then fused and fed to the attention stage for feature refinement and, finally, to the fully-connected layers for prediction. The internal architecture of the proposed model is further described in the following subsection.

### 2.1. CNN-LSTM

Dual CNN-LSTM architecture integrates CNN and LSTM for solar energy prediction. The proposed model has the ability to store the irregular complex trend and can extract complex features from historical solar power generation data. The first stream is incorporated to extract spatial features via CNN from the input data, while the second stream is responsible for temporal features extraction using LSTM. The CNN is a well-known deep learning architecture consisting of four types of layers, namely convolutional, pooling, fully connected, and regression layers [49]. The convolutional layers include multiple convolution filters which perform convolutional operations between convolutional neuron weights and input volume connected regions which generate a feature map [50,51]. The LSTM architecture is responsible for storing time information about important characteristics of solar power data. It supplies a solution by maintaining log-term memory by merging memory units that can update the previous hidden state [52]. With this function, it will be easier to understand temporal relationships in a long-term sequence. In this case, gate units receive the output values from the preceding CNN layer. The LSTM network addresses vanishing and explosive gradient problems that can happen when learning basic RNNs. The three gates unit’s mechanism can be used for determining the state of each individual memory cell. The input, output, and forget gates represent the gate unit. The mathematical of an LSTM from input to output generation is given in Equations (1)–(6).

(1) $${ƒ}_{t}=\Phi \;\left({Ŵ}_{f}\;\cdot \;\left[h_{t-1},\;x_{t}\right]+B_{f}\right)\;$$

(2) $$i_{t}=\Phi \;\left({Ŵ}_{i}\;\cdot \;\left[h_{t-1},\;x_{t}\right]+B_{i}\right)$$

(3) $${Ċ}_{t}=tanh\;\left({Ŵ}_{C}\;\cdot \;\left[h_{t-1},\;x_{t}\right]+B_{C}\right)$$

(4) $$C_{t}=f_{t}\;x\;C_{t-1}+i_{t}\;x\;{Ċ}_{t}\;$$

(5) $$o_{t}=\Phi \left({Ŵ}_{o}\;\cdot \;\left[h_{t-1},\;x_{t}\right]+B_{o}\right)$$

(6) $$h_{t}=o_{t}\;x\tanh(\Phi \left(C_{t}\right).$$

where $x_{t}$ is the input, hidden layer output is represented by $h_{t}$, Φ is the sigmoid function, and $C_{t}$ is the cell state, while its state candidate is represented by ${Ŵ}_{i}$, ${Ŵ}_{o}$, ${Ŵ}_{f}$, and ${Ŵ}_{C}$, which are the input, output, forget gate, and memory cells weights, respectively, while $B_{i}$, $B_{o}$, $B_{f}$, and $B_{c}$ are the bias terms for the input, output, forget gate, and cell, respectively. Finally, the output of CNN and LSTM streams are then fused with a concatenation layer and faded to attention layers for further processing.

### 2.2. Attention Mechanism

The final output of deep learning architectures named (CNN and LSTM) are integrated to obtain a single feature vector, and then fed the output streams to the self-attention SA mechanism to determine a representative feature vector for final forecasting. In addition, the invisible detail at different timestamps has a high impact on final results, but the CNN and LSTM streams are unable to predict forecasting accurately. To cope with these issues, our work is focused on integrating the SA architecture which has the capability to strengthen dominant and undermine trivial details by adaptively weighting the hidden features. In this paper, we utilized the SA architecture for the recognition of dominant features; in this regard, the combined feature vector of CNN and LSTM streams is used as an input to the SA network before forecasting. Moreover, the correlation of the proposed architecture at different timestamps among hidden features is investigated from every dimension. The calculation of the hidden features score, such as the $k^{th}$ timestamp and $N^{th}$ dimension, is based on Equation (7), as follows:

(7) $$S_{J,\;d}=f_{i}(w_{k,\;n}\left[h_{1,\;n},\;h_{2,\;n},h_{3,\;n},\ldots h_{n,\;k}\right),\;N=1,\;2,3\ldots n,\;k=1,2,3\ldots n_{i}$$

where $g_{k,\;n}$ indicates the $d^{th}$ dimension of the invisible state at $k^{th}$ timestamp, whereas the weight matrix, such as $w_{k,\;n}$, $f_{i}$ is a function applied using dense layers, and n and $n_{i}$ describe the number of timestamps and hidden feature dimensions, respectively.

The proposed network also contains dense layers, which are utilized to forecast power (PV) for a certain period of time, for instance an hour ahead of the PV power forecasting. The final output of the SA architecture is flattened to a $Z^{i}=z_{1}^{\;},\;z_{2}^{\;},\;z_{3}^{\;}\ldots .z_{n}^{\;}$ feature vector, whereas i represents the output dimensions of the proposed model. The output of the S-AM architecture is fed to the fully connected layers as an input, where the mathematical form of these layers is presented as follows in Equation (9):

(8) $$Z_{i}^{l}=\sum_{j}^{\;}w_{ji}^{l-1}(x\left(X_{\;i}^{l-1}\right)+b_{j}^{l-1}$$

where $w_{ji}^{l-1}$ indicates a weigh metric, x describes the activation function, namely the $X_{\;i}^{l-1}$ input data in this equation, while $B_{j}^{l-1}$ represents the bias term.

### 2.3. DSCLANet Archatecture

The architecture of DSCLANet includes CNN, LSTM, attention, and fully connected layers. Optimal DSCLANet architecture is developed by adjusting various parameters, including the size of the filter for CNN, the size of the kernel, the size of the LSTM cell, etc. Several experiments are conducted to choose the optimal parameters for the model before finalizing its internal parameters. The two streams allow for the parallel extraction of spatiotemporal features from large data sets, which are inputs to both streams. The CNN stream includes three CNN layers, while the LSMT stream includes two LSTM layers for each type of feature extraction. A concatenation layer is then applied to the output of both streams, followed by a feature-attentional layer and fully connected layers. The internal architecture of DSCLANet in terms of number of parameters, filters, and kernels is given in Table 2. In the first stream, the hyper-parameters of CNN layer 1 are as follows: the filter size is set to 32, with a kernel size of 5, padding is set to the same, the stride is set to 1, with default valid padding, and we used ReLU as the activation function. In the second CNN layer, the filter size is set to 64 with a kernel size of 3 while other hyper-parameters are the same as CNN layer 1. Furthermore, in the third CNN layer, the filter size is set to 128 while the kernel size of 1 is used. Other hyper-parameters of CNN layer 3 are the same as CNN layer 1. In the second stream, two LSTM layers are used with the same cell size of 100. These streams are then concatenated with a fusion layer, and the output is forwarded to the attention layer. The combined feature vector from both streams of the network includes redundant information, making the network computationally expensive, leading to non-convergence of the network, and achieving limited performance. Thus, the attention layer is used to enable the network to remove the redundant information and to enable the network to focus on important information while ignoring the rest of the information, which leads to fast convergence of the network and achieves considerable performance. This optimal feature is then passed to a fully connected layer for the final prediction, where 3 fully connected layers of sizes 64, 32, and 12 are used in DSCLANet.

## 3. Results

This section delivers a comprehensive discussion about evaluation metrics, datasets, and experimental results. The experiments are conducted in the Keras framework with a backend TensorFlow, utilizing a GeForce RTX 2070 graphics card.

### 3.1. Evaluation Metrics

The performance of the DSCLANet is assessed on standard evaluation metrics, such as MAE, MBE, RMSE, and MSE. These are common metrics used in the literature to evaluate the forecasting performance of solar power prediction models. The MAE is the average absolute difference between actual and predicted values, and MBE indicates the average difference between these values. The MSE is the square difference between predicted and actual data, while RMSE is the square root of MSE. The mathematical equation of these metrics is given in Equations (9)–(12), as follows:

(9) $$MAE=\frac{\sum_{n=1}^{m}\left|A_{n}-P_{n}\right|}{N}$$

(10) $$MBE=\frac{\sum_{n=1}^{m}{\left(P_{n}-A_{n}\right)}^{\;}}{N}$$

(11) $$MSE=\frac{\sum_{n=1}^{m}{\left(A_{n}-P_{n}\right)}^{2}}{N}$$

(12) $$RMSE=\sqrt{\frac{\sum_{n=1}^{m}{\left(A_{n}-P_{n}\right)}^{2}}{N}}$$

where A represents the actual and P represents the predicted values by the model.

### 3.2. Datasets

In this work, we utilized DKASC Alice Spring DKASC-AS datasets to evaluate the performance of the proposed and other models. Three datasets are selected from DKASC-AS, namely Trina 10.5 kW mono-Si Dual 2009 (Trina 1A), Trina 23.4 kW mono-Si Dual 2009 (Trina 1B), and eco-Kinetics 26.5 kW mono-Si Dual 2010 (Eco 2). These datasets include historical weather and solar power generation data with different generation capacities installed on different dates. Detailed information of the datasets, such as installation date, number of panels, type of panel, etc., are available of the DKASC website [53]. All the datasets are split into 70%, 20%, and 10% training, testing, and validation data, respectively. The proposed model and other ablation study models are evaluated using two-hour historical data as input to predict one hour ahead power generation.

### 3.3. Performance Evaluation of Deep Learning-Based Models

**Figure 2:**
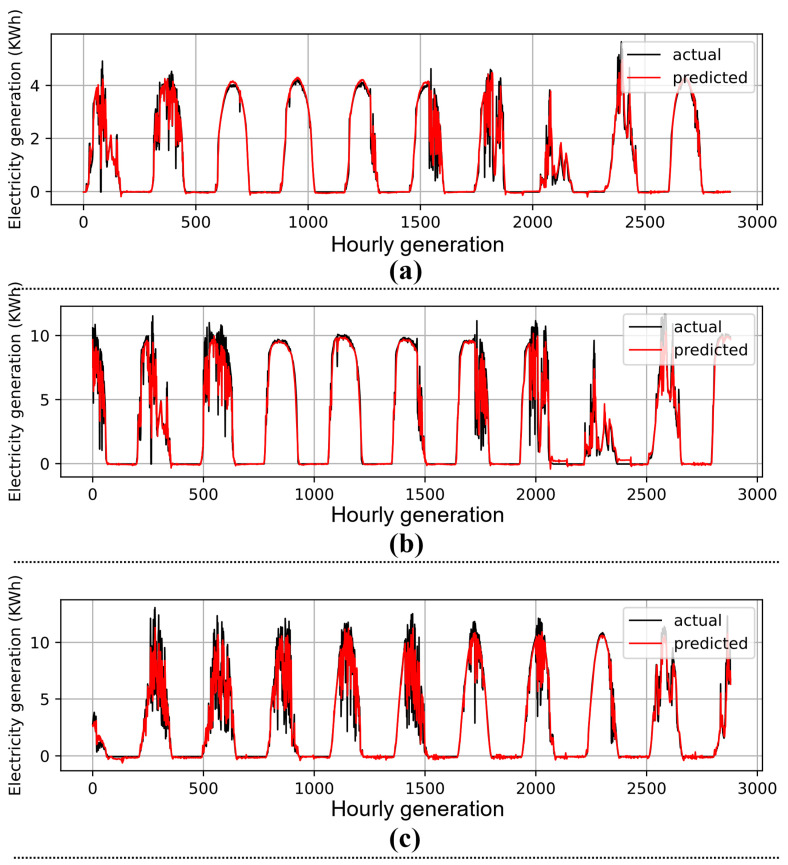
Prediction performance of DSCLANet with (**a**) dataset1, (**b**) dataset2, and (**c**) dataset 3.

To substantiate the robustness of the proposed DSCLANet, we conducted experiments on several models based on deep learning. These models include LSTM, CNN, GRU, CNNGRU, CNNLSTM, and DCNN-BRLSTM. The results attained by each model for every dataset is demonstrated in Table 3. For instance, LSTM achieved 0.0804 MSE, 0.143 MAE, and 0.2836 RMSE over the Trina 1A dataset, while these values were 0.0767, 0.1473, and 0.2769, and 0.0416, 0.1069, and 0.2041 over the Trina 1B and Eco 2 datasets, respectively. The CNN achieved 0.0699 MSE, 0.1526 MAE, and 0.3108 RMSE over the Trina 1A dataset, 0.1196 MSE, 0.2041 MAE, and 0.3458 RMSE over the Trina 1B dataset, and 0.0433 MSE, 0.1288 MAE, and 0.2081 for the RMSE Eco 2 dataset. Furthermore, GRU attained 0.0848 MSE, 0.1518 MAE, and 0.2912 RMSE over the Trina 1A dataset, 0.065 MSE, 0.1196 MAE, and 0.2549 RMSE over the Trina 1B dataset, and 0.0384 MSE, 0.1011 MAE, and 0.196 RMSE over the Eco 2 dataset. Compared to the output of these models’ hybrid models, such as CNNGRU and CNNLSTM, DSCLANet achieved better prediction results due to learning both spatiotemporal information from historical data. For instance, CNNLSTM achieved 0.0679 MSE, 0.12 MAE, and 0.2606 RMSE over the Trina 1A dataset, 0.0648 MSE, 0.131 MAE, and 0.2546 RMSE over the Trina 1B dataset, and 0.0298 MSE, 0.088 MAE, and 0.1725 RMSE over the Eco 2 dataset. Similarly, CNNGRU achieved (0.0793, 0.01519, and 0.2817), (0.0641, 0.1365, and 0.2531), and (0.032, 0.0879, and 0.1789) values for the Trina 1A, Trina 1B, and Eco 2 datasets, respectively. The proposed DSCLANet further reduces the error metrics and achieved the lowest error rate as compared to the abovementioned models. The proposed DSCLANet achieved 0.0167 MSE, 0.0632 MAE, and 0.1291 RMSE over the Trina 1A dataset, 0.0279 MSE, 0.0889 MAE, and 0.167 RMSE over the Trina 1B dataset, and 0.0074 MSE, 0.0479 MAE, and 0.0858 RMSE over the Eco 2 dataset. Furthermore, the actual and predicted results of DSCLANet over each dataset are given in Figure 2.

### 3.4. Comparison with State-of-the-Art

In this section, we compared the performance of DSCLANet with other baselines. The performance of the proposed approach is compared with the wavelet packet decomposition (WPD-LSTM) [54], RCC-LSTM [55], HIMVO-SVM [56], ESN-CNN [7], CNN-LSTM [57], DenseNet [28], LSTM-CNN [48], ELM [58], graph-network [59], and SolarNet [60] models. The detailed performance of these models is given in Table 4, where the DSCLANet attained the smallest error rates comparatively. The DKASC Alice Spring sites include several solar power plants, and the researcher evaluated their model performance over one, two, or three sites’ data. Therefore, in this work, we compared the average performance of DSCLANet for three sites’ data, namely Trina 1A, Trina 1B, and Eco 2, with these methods. Comparatively, the DSCLANet achieved a better performance in all error metrics, as shown in Table 4.

## 4. Conclusions

It is important to forecast solar power generation accurately to avoid penalties from customers, build trust in the energy markets, and schedule power generation. In mainstream deep learning and traditional learning methods, features are based on simple phenomena, and they only take into account spatial or temporal features to get around the nonlinearities of solar power generation series. However, some studies combine different methods for spatial and temporal feature extraction via a stacked layers mechanism. Therefore, in this work, we developed a dual-stream CNN-LSTM network for solar power prediction. The performance of DSCLANet is evaluated for real solar power datasets collected from a photovoltaic system located in Alice Springs, Australia. Before selecting the proposed model, extensive experiments are performed over different deep learning-based models. Furthermore, we compared the performance of the DSCLANet with other baselines and found that the proposed model outperforms them in terms of error reduction. Alongside higher performance, the DSCLANet uses two architectures, namely LSTM and CNN, for spatial and temporal feature extraction. However, combining multiple methods for spatial and temporal feature extraction increases the model complexity. Therefore, in the near future, we intend to develop a solo architecture with the ability to extract both types of features. Furthermore, we also intend to investigate emerging technologies, such as probabilistic forecasting, incremental learning, active learning, and reinforcement learning for solar power prediction.

## Figures and Tables

**Table 2:** Internal architectures of DSCLANet.

Type	No. of Filters	Kernel-Size	Params
Conv	32	5	992
Conv	64	3	6208
Conv	128	1	24,704
LSTM (100)	-	-	44,400
LSTM (100)	-	-	80,400
Fusion	-	-	-
Attention	-	-	1089
Dense_64	-	-	4128
Dense_32	-	-	12,928
Dense_12	-	-	396

**Table 3:** Performance comparison of several models developed during the ablation study.

Dataset	Method	MSE	MAE	RMSE
Trina 1A	CNN	0.0966	0.1526	0.3108
	LSTM	0.0804	0.143	0.2836
	GRU	0.0848	0.1518	0.2912
	CNNLSTM	0.0679	0.12	0.2606
	CNNGRU	0.0793	0.1519	0.2817
	DSCLANet	0.0167	0.0632	0.1291
Trina 1B	CNN	0.1196	0.2041	0.3458
	LSTM	0.0767	0.1473	0.2769
	GRU	0.065	0.1196	0.2549
	CNNLSTM	0.0648	0.131	0.2546
	CNNGRU	0.0641	0.1365	0.2531
	DSCLANet	0.0279	0.0889	0.167
Eco 2	CNN	0.0433	0.1288	0.2081
	LSTM	0.0416	0.1069	0.2041
	GRU	0.0384	0.1011	0.196
	CNNLSTM	0.0298	0.088	0.1725
	CNNGRU	0.032	0.0879	0.1789
	DSCLANet	0.0074	0.0479	0.0858

**Table 4:** Performance comparison of several models developed during the ablation study.

Method	MSE	MAE	RMSE
WPD-LSTM [54]	-	-	0.2357
RCC-LSTM [55]	-	0.587	0.94
HIMVO-SVM [56]	-	-	2805
ESN-CNN [7]	0.0309	0.0971	0.1731
CNN-LSTM [57]	-	0.126	0.343
DenseNet [28]	0.081	0.152	-
LSTM-CNN [48]	-	0.221	0.621
ELM [58]	-	0.2367	-
Graph-network [59]	-	0.117	0.336
SolarNet [60]	-	0.175	0.309
**DSCLANet**	**0.0173**	**0.0667**	**0.1273**

## Data Availability

Not applicable.
